# Effects of exercise dose based on the ACSM recommendations on patients with post-stroke cognitive impairment: a systematic review and meta-analyses

**DOI:** 10.3389/fphys.2024.1364632

**Published:** 2024-06-03

**Authors:** Xuejiao Zhao, Juan Li, Chao Xue, Yujie Li, Ting Lu

**Affiliations:** ^1^ School of Nursing, Guizhou University of Traditional Chinese Medicine, Guiyang, Guizhou, China; ^2^ Department of Nursing, Guizhou Provincial People’s Hospital, Guiyang, Guizhou, China

**Keywords:** exercise, American college of sports medicine, post-stroke cognitive impairment, dose, meta-analysis

## Abstract

**Purpose:**

This review aimed to assess the impact of different exercise dosages on cognitive function in individuals with post-stroke cognitive impairment (PSCI).

**Methods:**

Four electronic databases—Embase, PubMed, Web of Science, and Cochrane Library—were systematically searched from inception to 01 January 2024, focusing on the impact of exercise therapy on cognitive function in individuals with PSCI. Only randomized controlled trials meeting the criteria were included. The exercise therapy dose and adherence were evaluated following the American College of Sports Medicine (ACSM) guidelines, categorized into a high compliance group with ACSM recommendations and a low or uncertain compliance group. A random-effects model compared the effect of ACSM compliance on cognitive function in individuals with PSCI, with the effect size represented by the standardized mean difference (SMD) and a 95% confidence interval (CI).

**Results:**

In total, 18 studies meeting the criteria were included, with data from 1,742 participants. The findings suggested a beneficial effect of exercise on cognitive function in individuals with PSCI [SMD = 0.42, 95% CI (0.20, 0.65)]. Ten studies were categorized as the “high adherence group” and eight in the “low or uncertain adherence group” based on the ACSM recommendations. The subgroup analysis revealed that the SMD of the high compliance group was 0.46 (95% CI: 0.10, 0.82) (*p* = 0.01), while the SMD of the low or uncertain compliance group was 0.38 (95% CI: 0.07, 0.70) (*p* = 0.02).

**Conclusion:**

Our study indicates the beneficial impact of exercise for patients with PSCI over no exercise. Furthermore, high adherence to the exercise dose recommended by ACSM guidelines demonstrated a more substantial improvement in cognitive function than low or uncertain adherence in patients with PSCI.

**Systematic Review Registration:** https:// www.crd.york.ac.uk/prospero/#myprospero, identifier CRD42023487915.

## 1 Introduction

Post-stroke cognitive impairment (PSCI) stands out as a major sequela in patients after a stroke. It encompasses a range of cognition-related clinical syndromes, spanning from mild cognitive impairment to dementia, manifesting within 3–6 months following a stroke event (Sun et al., 2014; Koton et al., 2022; Rost et al., 2022). Patients with PSCI may grapple with impairments in diverse cognitive areas, including attention, memory, delayed memory, executive function, calculation, and visuospatial dysfunction, among others (Zhang and Bi, 2020; Kernan et al., 2021; Rost et al., 2022). Regrettably, PSCI tends to be overlooked compared to the more apparent physical disabilities (Liu et al., 2023). The overall prevalence of PSCI fluctuates between 20% and 80% (Sun et al., 2014; Ding et al., 2019). A recent multicenter cross-sectional study disclosed that the incidence of PSCI among Chinese individuals with a first-ever stroke was 78.7% (He et al., 2023). Notably, PSCI affects over one-third of stroke survivors, potentially leading to adverse clinical consequences (Mijajlović et al., 2017). It not only heightens the challenges of overall stroke patient rehabilitation, diminishes the quality of life, but also contributes to higher rates of disability and mortality, increased readmission rates, and ultimately imposes a substantial financial burden on caregivers and healthcare providers (Atteih et al., 2015; Obaid et al., 2020; Zhang and Bi, 2020). Consequently, early screening and management of PSCI emerge as urgent priorities.

Presently, PSCI treatment methods encompass pharmacological therapy (such as cholinesterase inhibitors: galantamine, rivastigmine, or donepezil) and non-pharmacological therapy (including cognitive training, exercise intervention, the motor-cognitive dual task training, acupuncture therapy, or lifestyle interventions, etc.) (Quinn et al., 2021). However, the long-term efficacy of pharmacological interventions remains uncertain, patient tolerance varies, and specific medication treatments may induce serious adverse effects, such as hepatotoxicity and gastrointestinal discomfort (Narasimhalu et al., 2010; Battle et al., 2021). Therefore, non-pharmacological interventions have garnered widespread attention. Exercise, as a crucial non-drug therapy, can enhance the quality of life in the general population and benefit the cardiovascular system and cognitive function in individuals after a -stroke (Huang et al., 2022).

The 2019 Canadian Stroke Best Practice Guidelines propose that exercise training can serve as an additional treatment method for cognitive deficits, encompassing various aspects like memory, attention, and executive performance (Lanctôt et al., 2020). Recent systematic reviews and meta-analyses reveal that exercise treatment not only enhances cognitive function but also improves motor function and activities of daily living in patients with PSCI (Li et al., 2023; Zhang et al., 2023). Hernandez and Gonzalez-Galvez (2021) synthesized the original studies on the impact of physical exercise programs on cognitive function in patients who suffered from a stroke to identify the best frequency, length, and type of program. They found that physical exercise programs were superior to rehabilitation programs for improving cognitive function. The recommended dose is at least 30 min per session, three sessions per week for at least 6 weeks. It is worth mentioning that motor-cognitive dual task training, a non-pharmacological intervention that combines motor tasks with cognitive tasks, has shown promise in reducing cognitive decline and improving quality of life in individuals with PSCI (Kim et al., 2014). However, since the primary focus of this study is to explore the optimal dosage of exercise therapy, studies that solely focus on motor-cognitive dual task training without a cognitive training control group should be excluded from this analysis. Conversely, studies that primarily involve motor-cognitive dual task training along with a cognitive training control group are suitable for inclusion in this review. Currently, numerous studies have confirmed the effectiveness of exercise on cognition for individuals with PSCI. Nevertheless, there remains a scarcity of research regarding the appropriate exercise dosage. Determining how to standardize exercise interventions to make them more systematic and reproducible remains a significant challenge. Therefore, further investigations are warranted to delve into the optimal exercise dose for patients with PSCI.

The American College of Sports Medicine (ACSM) recommends a regular land-based exercise program for most adults, which includes resistance, cardiopulmonary, flexibility, and neuromotor training. The ACSM also provides detailed exercise dosages, providing an important foundation for developing standardized exercise regimens (Garber et al., 2011). Chang et al. (2015) conducted exercise therapy in healthy young men following the ACSM guidelines and found that a session consisting of a 5-min warm-up, 20 min of moderate-intensity exercise, and a 5-min cooldown improved cognition. In contrast, shorter or longer durations of moderate exercise yielded negligible benefits. Currently, despite the increasing number of studies on exercise intervention for patients with PSCI, there is a lack of research evaluating exercise doses according to ACSM guidelines. As a result, the impact of varying exercise dosages on the cognition of PSCI patients is still debated. Therefore, further studies are needed to delve into the optimal exercise dosage for patients with PSCI. The objective of this study is to validate the efficacy of exercise in improving cognition and juxtapose the consequences of high and low/uncertain compliance following ACSM guidelines among patients with PSCI, to provide evidence for the development of more standardized, reproducible and effective exercise prescription.

## 2 Methods

This review strictly adhered to the guidelines outlined in the Preferred Reporting Items for Systematic Reviews and Meta-Analyses (PRISMA) (Page et al., 2021) and was duly registered in PROSPERO (CRD42023487915).

### 2.1 Search strategy

A comprehensive search encompassed electronic databases—Embase, PubMed, Web of Science, and Cochrane Library—from their inception to 01 January 2024. The search strategy was meticulously constructed in accordance with the PICOS principle: (P) Population—patients with PSCI; (I) Intervention—various land-based exercises; (C) Comparator—limited to conventional care or routine physiotherapy (inclusive of usual balance and stretching training); (O) Outcomes—cognitive function tests specifically for patients with PSCI; (S) Study design—Clinical randomized controlled trials (RCTs). Using PubMed as an example, Table 1 presents the detailed summary of the search strategy. This search involved a combination of subject terms and free words. Furthermore, in order to obtain additional potential researches, we meticulously screened the reference lists of included studies and pertinent reviews. As necessary, we communicated with authors to acquire supplementary information.

**TABLE 1:** Search strategy on PubMed.

#1	“stroke”[MeSH]
#2	(((((stroke [Title/Abstract]) OR strokes [Title/Abstract]) OR Acute Cerebrovascular Accident [Title/Abstract]) OR Apoplexy [Title/Abstract]) OR Brain Vascular Accident [Title/Abstract]) OR Cerebrovascular Accident [Title/Abstract]
#3	#1 OR #2
#4	“Cognitive Dysfunction”[MeSH]
#5	(((((((((Cognitive Dysfunction [Title/Abstract]) OR Cognitive Deficit [Title/Abstract]) OR Cognitive Decline [Title/Abstract]) OR Cognitive Disorder [Title/Abstract]) OR Cognitive Impairment [Title/Abstract]) OR Mental Deterioration [Title/Abstract]) OR Decline, Cognitive [Title/Abstract]) OR Disorder, Cognitive [Title/Abstract]) OR Impairment, Cognitive [Title/Abstract]) OR Dysfunction, Cognitive [Title/Abstract]
#6	#4 OR #5
#7	#3 AND #6
#8	(post-stroke cognitive impairment [Title/Abstract]) OR (cognitive impairment after stroke [Title/Abstract])
#9	#7 OR #8
#10	(((((((“Exercise"[MeSH]) OR “Resistance Training"[MeSH]) OR “Plyometric Exercise”[MeSH]) OR “Exercise Movement Techniques"[MeSH Terms]) “Exercise Therapy"[MeSH]) OR “Endurance Training”[MeSH]) OR″Tai Ji”[MeSH]) OR “Yoga”[MeSH])
#11	((((((((((((Exercise [Title/Abstract]) OR Resistance Training [Title/Abstract]) OR Plyometric Exercise [Title/Abstract]) OR Exercise Movement Techniques [Title/Abstract]) OR Exercise Therapy [Title/Abstract]) OR Endurance Training [Title/Abstract]) OR Tai Ji [Title/Abstract]) OR Yoga [Title/Abstract]) OR Qigong [Title/Abstract]) OR Baduanjin [Title/Abstract] OR Yijinjing [Title/Abstract]) OR Aerobic Exercises [Title/Abstract]) OR Physical Training [Title/Abstract]
#12	#10 OR #11
#13	#9 AND #12

### 2.2 Inclusion and exclusion criteria

Inclusion criteria: (1) Study subjects (aged 18 years and above) with a definitive diagnosis of ischemic or hemorrhagic stroke, with or without confirmed cognitive dysfunction, were considered for inclusion. Mixed studies were also included when data on patients who suffered from a stroke could be extracted separately; (2) The experimental group received any land-based exercise treatment, comprising resistance training, flexibility training, aerobic training, and mixed training combined with multiple exercises; (3) The control group received routine care, conventional physiotherapy, health education, or no treatment; (4) Outcome indicators focused on overall cognitive function or ≥1 domain-specific aspect of cognition, such as memory, delayed memory, attention, executive function, and calculation; and (5) RCTs.

Exclusion criteria: (1) Exclusion of animal research; (2) Reports, protocols, reviews, case reports, and conference papers, among others; (3) Exclusion of any studies involving aquatic exercise; (4) Failure to obtain the full text; (5) Elimination of duplicate publications; (6) Data that could not be extracted; (7) Exclusion of studies falling outside the RCT category; (8) Exclusion of studies where exercise served as the control group.

### 2.3 Study selection

Endnote software facilitated the screening and management of all literature. Initially, two authors (XJZ and YJL) independently scrutinized titles and abstracts, excluding duplications, reviews, conference abstracts, correspondence, case reports, protocols, animal studies, and non-RCTs. Subsequently, two researchers independently re-evaluated abstracts to determine inclusion and exclusion criteria. Finally, two researchers conducted a thorough and independent evaluation of all the remaining articles by carefully examining the contents of each article to ascertain the final included literature. In case of any discrepancies or differences in opinion, the researchers engaged in discussions and sought the assistance of a third author (CX) to reach a final decision. Importantly, there were no restrictions imposed on publication dates or languages.

### 2.4 Data extraction

Two researchers (XJZ and TL) independently extracted pertinent data using a standardized and pre-designed form. This encompassed publication characteristics (title, first author, year of publication, and country), methodological approach (group design, sample size, and intervention measures), exercise intervention specifics (type, time, frequency, duration, length, intensity, repetition, and set), subjects’ characteristics (age and sex ratio), and outcome features. In the case of studies with multiple follow-ups, we exclusively extracted data immediately after the intervention.

Subsequently, two raters (XJZ and TL) independently evaluated the dose (including frequency, intensity, workload, duration, etc.) and adherence to exercise interventions in patients with PSCI following ACSM guidelines (Garber et al., 2011) (Table 2). Each exercise indicator was assessed on a 2-point scale: 2 points indicated meeting the criteria, 1 point as uncertain, and 0 point as not meeting the criteria. Using this grading system, we computed the adherence to ACSM recommendations for exercise dose in each study. If the proportion was 75% or higher, it was considered to demonstrate high compliance with ACSM recommendations. Conversely, if the proportion was less than 75%, it was categorized as indicative of low or uncertain compliance with ACSM recommendations. In cases of any disagreement, the third reviewer (CX) participated in discussions to reach a consensus.

**TABLE 2:** The ACSM recommendations for cardiorespiratory fitness, muscular strength and flexibility in apparently healthy adults.

Exercise dose	Cardiorespiratory exercise	Resistance exercise	Flexibility exercise
Frequency	3–5 days per week	2–3 days per week	≥2–3 days per week, daily
Intensity/workload	40%–60% VO_2_R or HRR RPE of 12–13 on a 6–20 scale	Start with 40%–50% 1RM, more capable with 60%–70% 1RM	Stretch until you feel your muscles being pulled tight or a slight discomfort
Duration	Continuous or cumulative 30 min	≥1 group, 8–12 repetitions	Keep static pulling for 10–30 s; repeat 2–4 times

Note: VO_2_ R, oxygen uptake reserve. HRR, heart rate reserve. RPE: ratings of perceived exertion.

### 2.5 Risk of bias of individual studies

Two raters (YJL and TL) independently assessed the methodological quality of included studies using the Cochrane Bias Risk Assessment Tool for RCTs. Seven domains were considered: (1) randomized sequence generation, (2) allocation concealment, (3) blinding of participants and personnel, (4) blinding of outcome assessment, (5) incomplete outcome data, (6) selective reporting, and (7) other bias. Trials were categorized into three levels of risk of bias: low risk, high risk, and unclear risk (no reporting or missing information).

### 2.6 Data analysis

Meta-analyses were conducted using Review Manager 5.4 for comparing the results of the included studies. In studies where exercise was the intervention, all variables were continuous and reported as means with standard deviation (SD).

Subgroup analyses of high and low or uncertain compliance groups were performed. The heterogeneity among studies within each subgroup was assessed using the Higgins *I* ^2^ statistic, as suggested by the Cochrane Handbook. The level of heterogeneity was categorized as low (25% < *I* ^2^ ≤ 50%), moderate (50% < *I* ^2^ ≤ 75%), or high (*I* ^2^ > 75%) (Higgins et al., 2003). In cases where *I* ^2^ ≤ 50%, a fixed-effect model was employed to determine the effect size; conversely, if *I* ^2^ > 50%, a random-effects model was utilized to estimate the effect size, indicated by the standardized mean difference (SMD) along with a 95% confidence interval (95% CI).

## 3 Results

### 3.1 Study and identification and selection

**FIGURE 1:**
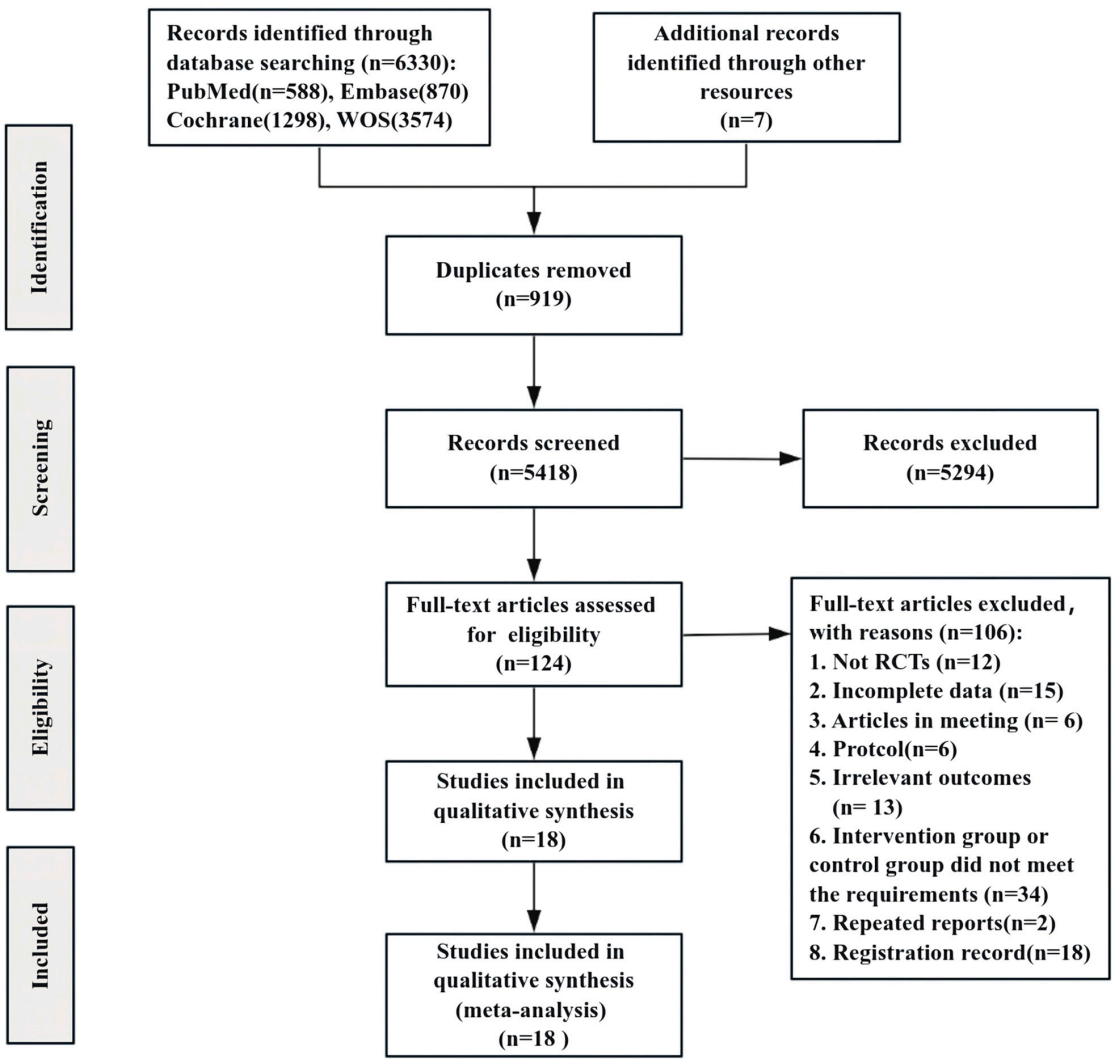
Flow chart of literature selection.

A total of 6,330 studies were initially identified through the search strategy, with an additional 7 documents manually sourced from other resources. We screened 5,418 unique records to eliminate duplicates and excluded 5,294 documents after reviewing the titles and abstracts, resulting in 124 records. Following a comprehensive examination of the entire text, 106 articles were further excluded, ultimately incorporating 18 studies in this review (Figure 1).

### 3.2 Quality assessment of the included studies

**FIGURE 2:**
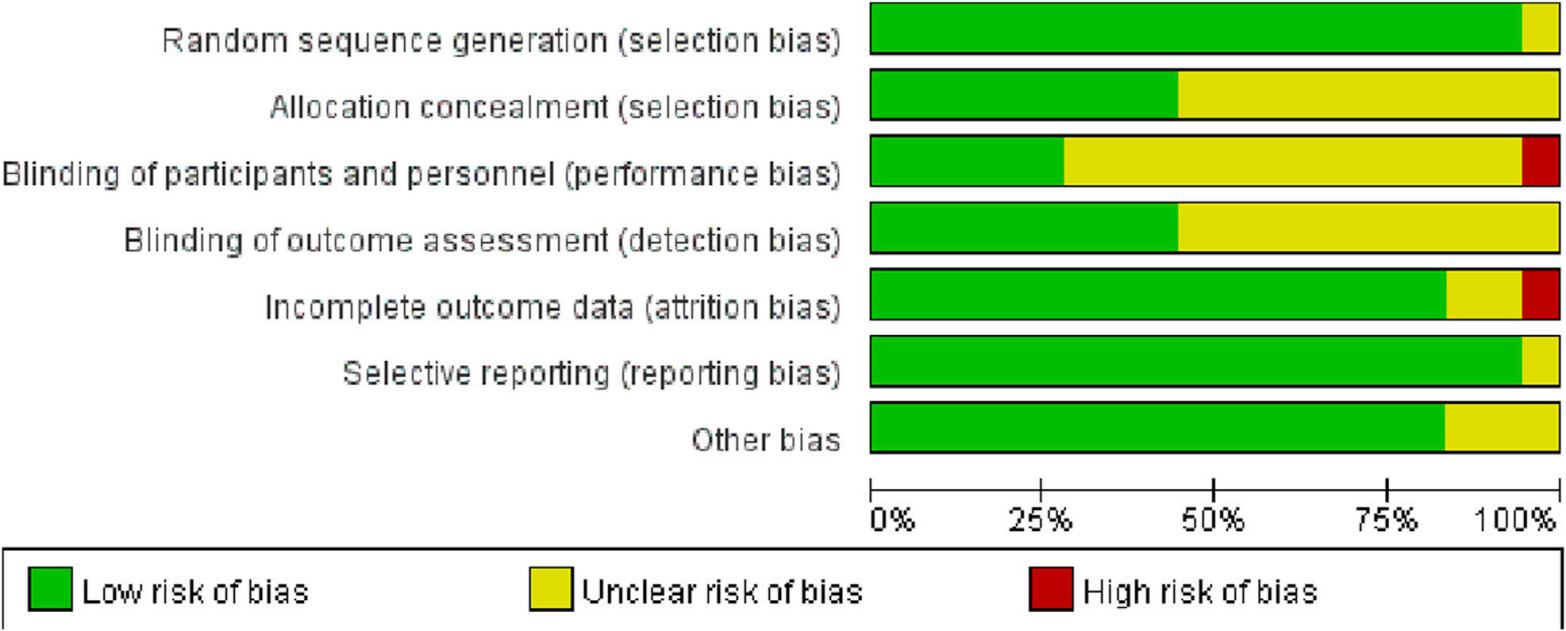
Combined percentage risk of bias in each risk domain for all included trials.

**FIGURE 3:**
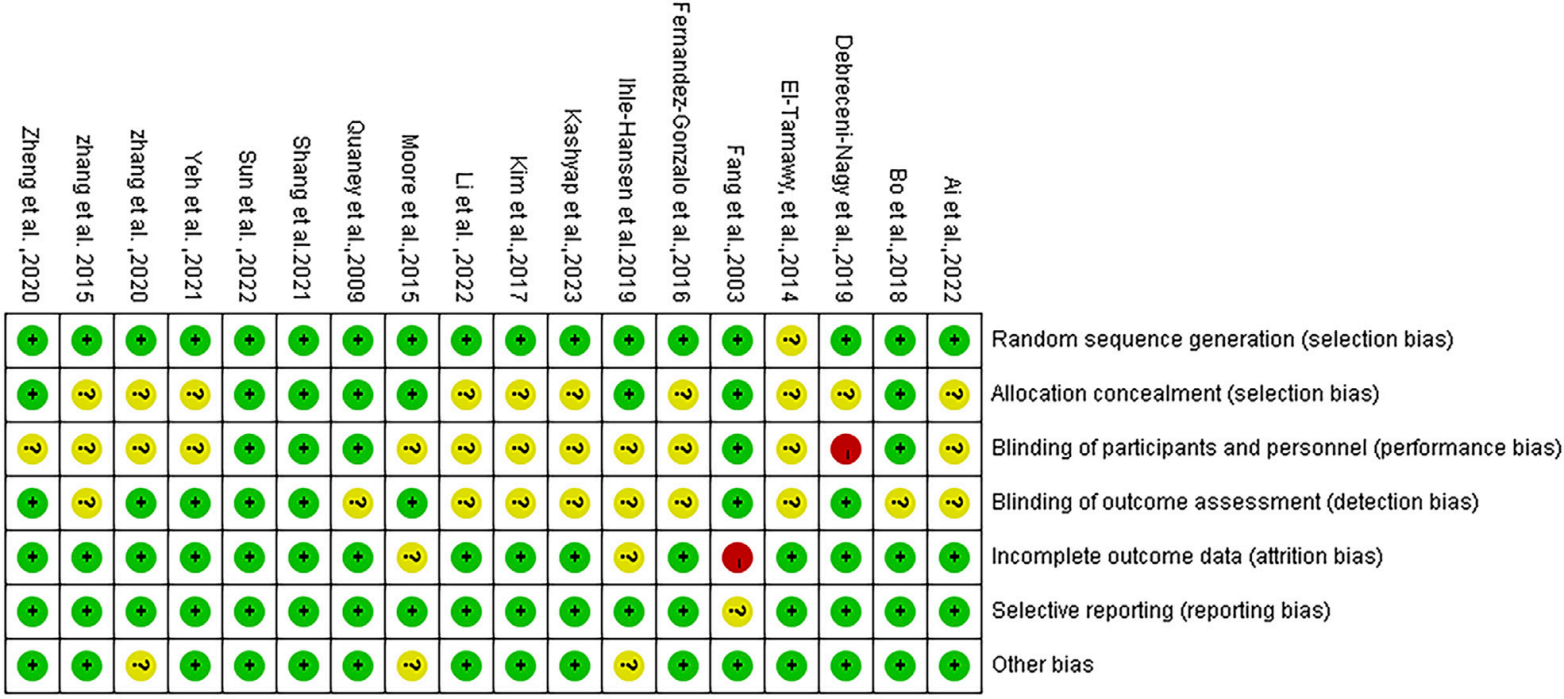
Risk of bias summaries for all exercise trials.

Following methodological quality evaluation of the included studies, it was determined that the evaluation consistency rate was 78%. Specifically, 14 studies exhibited consistent evaluation results, while 4 studies showed inconsistent results. As a result, a third author was consulted to reach a final decision. According to the Cochrane bias risk assessment tool in RCTs, 17 studies detailed the method of random sequence generation, 8 explained the allocation concealment, 5 explicitly described the blinding of subjects and intervention implementers, and 8 explicitly defined the blinding of outcome evaluators. Only one study exhibited incomplete outcome data and did not specify the method for handling missing values. The detailed assessment of literature quality is depicted in Figures 2, 3.

### 3.3 Characteristics of the included studies

Table 3 provides an overview of the specific features regarding the studies that were included. A total of 18 studies comprised a participant pool of 1,742 individuals, with an average age of 61 years (range: 49–72 years, approximately 41% female). The experimental group comprised 860 participants, while the control group had 882 participants. The RCTs examined sample sizes ranging from 29 to 420 participants. The majority of the included studies utilized the Montreal Cognitive Assessment or Mini-Mental State Examination scale to evaluate cognitive function. However, a few studies employed the Addenbrooke’s Cognitive Examination-Revised, Trail Making Test, or Stroop test scale. The primary intervention duration varied from 4 weeks to 18 months, and the frequency of exercise sessions varied from 2 to 7 days every week. Regarding the classification of interventions, 14 studies focused on cardiorespiratory exercise, 10 studies concentrated on resistance exercise, and 3 studies explored flexibility exercise (Table 4).

**TABLE 3:** Characteristics of the studies included in the meta-analysis.

Author	Country	Year	Population	Age (mean ± SD)	Total/male/female	Intervention	Control	Outcome
El-Tamawy et al	Egypt	2014	patients with PSCI	T:48.4(6.39) C:49.67(6.98)	T:15/11/4 C:15/10/5	Aerobic exercises Length of Intervention: 8 weeks Freq: 3 times a week Duration: 40–45 min	Physiotherapy program	ACER
Zheng et al	China	2020	patients with PSCI	T:61.63(9.21) C:62.75(6.41)	T: 24/19/5 C: 24/22/2	Baduanjin exercise training Length of Intervention: 24 weeks Freq: 3 times a week Duration: 40 min	CON	MoCA
Kashyap et al	India	2023	patients with PSCI	T:52.85(13.70) C:55.18 (13.24)	T: 40/28/12 C: 40/NR/NR	Yoga Length of Intervention: 24 weeks Freq: 4–5 times a week Duration: 60 min	CON	MoCA
Sun et al	China	2022	patients with PSCI	T: 54.0 (14.0) C: 60.5 (10.6)	T: 17/12/5 C: 16/13/3	Motor training + CT Length of Intervention: 4 weeks Freq: 5 times a week Duration: 40 min	CT	MoCA
Yeh et al	Taiwan	2021	patients with PSCI	T: 53.05(14.53) C:60.17(12.13)	T: 20/12/8 C: 18/13/5	Aerobic exercise training + CT Length of Intervention: 12 weeks Freq: 3 times a week Duration: 30 min	CT	MoCA
Bo et al	China	2018	stroke patients with vascular Cognitive impairment	T: 65.12(2.56) C:64.36(2.31)	T: 42/23/19 C: 47/27/20	Physical exercise Length of Intervention: 12 weeks Freq: 3 times a week Duration: 50 min	CON	TMT-B ST DST MRT
Kim et al	Korea	2017	Stroke patients with cognitive function disorder	T: 50.71(14.81) C:51.87(17.42)	T: 14/9/5 C: 15/10/5	Exercise protocol for handgrip strength and walking speed Length of Intervention: 6 weeks Freq: 5 times a week Duration: 30 min	CON	MoCA
Zhang et al	China	2015	patients with cognitive impairment no dementia	T:68.63(11.35) C:68.81(11.57)	T: 220/124/96 C: 200/117/83	Aerobic combined with resistance exercise Length of Intervention: 12 weeks Freq: aerobics - 5 times/week; resistance training - 3times/week Duration: aerobics - 40 min; resistance training - 20 min	CON	MoCA
Fernandez-Gonzalo et al	Sweden	2016	Stroke patients (≥6 months post-stroke)	T: 61.2 (9.8) C: 65.7 (12.7)	T: 14/11/3 C: 15/11/4	Lower extremity resistance training Length of Intervention: 12 weeks Freq: 2 times a week Duration: 4 sets of 7 repetitions	CON	DST ST TMT RAVLT
Ihle-Hansen et al	Norway	2019	patients with first ever or recurrent stroke	T: 71.4 (11.3) C: 72.0 (11.3)	T: 177/99/78 C: 185/120/65	Physical activity Length of Intervention: 72 weeks Freq: 7 times a week Duration: 30 min	CON	MMSE
Fang et al	China	2003	Stroke patients	T: 65.49 (10.94) C: 61.8 (10.94)	T: 50/33/17 C: 78/44/34	Bobath techniques and passive movements training of the affected limb Length of Intervention: 4 weeks Freq: 5 times a week Duration: 45 min	CON	MMSE
Moore et al	UK	2015	Stroke patients (≥6 months post-stroke)	T: 68(8) C: 70(11)	T: 20/18/2 C: 20/16/4	Structured Exercise Intervention Length of Intervention: 19 weeks Freq: 3 times a week Duration: 45–60 min	stretching	ACER
Debreceni-Nagy et al	Hungary	2019	stroke patients	T: 59 (9.63) C: 62(11.48)	T: 19/13/6 C: 16/11/15	Aerobic training by cycle ergometer Length of Intervention: 4 weeks Freq: 5 times a week Duration: 30 min	CON	FIM DST
Quaney et al	USA	2009	Stroke patients (≥6 months post-stroke)	T: 64.10 (12.30) C: 58.96(14.68)	T: 19/10/9 C: 19/7/12	Progressive resistance aerobic exercise Length of Intervention: 8 weeks Freq: 3 times a week Duration: 45 min	stretching	ST TMT SRTT
Shang et al	China	2021	patients with acute ischemic stroke	T: 63.68(9.10) C: 64.13 (9.21)	T: 37/21/16 C: 39/20/19	Intensive grip training Length of Intervention: 12 weeks Freq: 3 times a week Duration: 50 min	CON	MoCA
Li et al	China	2022	patients with PSCI	T: 60.23(6.57) C:59.15(7.11)	T: 49/27/22 C: 52/32/20	Aerobic exercise Length of Intervention: 8 weeks Freq: 5 times a week Duration: 40–50 min	CON	MoCA
Ai et al	China	2022	patients with PSCI	T: 62.03(5.14) C: 63. 25 (5.59)	T: 58/39/19 C: 58/36/22	Resistance training + CT Length of Intervention: 8 weeks Freq: 5 times a week Duration: 30 min	CT	MoCA
Zhang et al	China	2020	stroke patients	T: 63.96(7.84) C: 63.75(7.96)	T: 69/41/28 C: 69/40/29	Aerobic exercise Length of Intervention: 12 weeks Freq: 4 times a week Duration: 20–30 min	CON	MMSE

Note: SD: standard deviation, T: experimental group, C: control group, CON: control group with conventional care (no exercise), CT: cognitive training, ACER: Addenbrooke’s Cognitive Examination-Revised, MoCA: montreal cognitive assessment, MMSE: mini mental state examination, TMT: trail making test, ST: stroop test, DST: digit span test, MRT: mental rotation test, RAVLT: the rey auditory verbal learning test, FIM: functional independence measure, SRTT: serial reaction timed task; NR, not reported, Freq: frequency.

**TABLE 4:** Exercise interventions evaluated according to the ACSM recommendations.

Author, year	Cardiorespiratory exercise						Resistance exercise								Flexibility exercise						ACSM adherence
	Freq		Intensity/workload		Duration		Freq		Intensity/workload		Repetitions		Sets		Freq		Intensity/workload		Duration		
	3–5 days per week		40%–60% VO_2_R or HRR; 64%–76% HRmax; RPE of 12–13 on a 6–20 scale		Continuous or cumulative 30 min		2–3 days per week		Start with 40%–50% 1RM, more capable with 60%–70% 1RM		8–12		≥1 group		≥2–3 days per week, daily		Stretch until you feel your muscles being pulled tight or a slight discomfort		keep static pulling for 10–30 s; repeat 2–4 times		Points (Percent)
Ai et al. (2022)							5	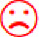	NR	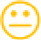	NR	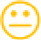	NR	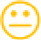							3/8(38%)
Bo et al. (2019)	3	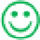	RPE:13–15	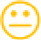	50 min	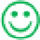	3	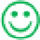	NR	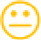	NR	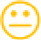	NR	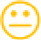	3	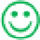	NR	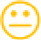	5 min	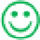	15/20(75%)
Debreceni-Nagy et al. (2019)	5	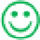	HRR: 40%–60%	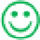	30 min	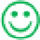															6/6(100%)
El-Tamawy et al. (2014)	3	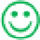	NR	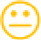	40–45 min	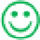															5/6(83%)
Fang et al., 2003	5	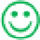	Passive movement training	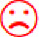	45 min	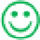															4/6(67%)
Fernandez-Gonzalo et al. (2016)							2	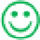	Maximum Repeat	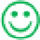	7	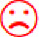	4	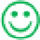							6/8(75%)
Ihle-Hansen et al. (2019)	7	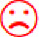	2–3 bouts of vigorous activity	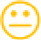	30 min	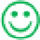															3/6(50%)
Kashyap, et al. (2023)															4–5	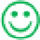	yoga practice intensity	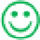	60 min	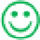	6/6(100%)
Kim and Yim (2017)	5	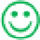	NR	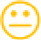	20 min	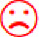	5	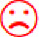	Ind. tail	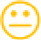	15	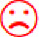	3	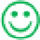							6/14(43%)
Moore et al. (2015)	3	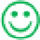	from the initial 40%–50% gradually increased to 70%–80% HRmax	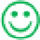	45–60 min	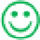	3	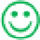	NR	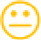	NR	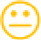	NR	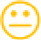							11/14(79%)
Quaney et al. (2009)	3	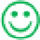	HRmax:70%	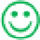	45 min	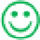	3	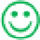	NR	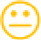	NR	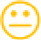	NR	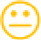							11/14(79%)
Shang et al. (2021)							3	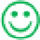	maximum strength	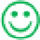	NR	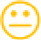	1–5	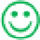							7/8(88%)
Sun et al. (2022)	5	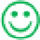	NR	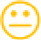	40 min	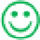	5	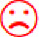	NR	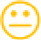	NR	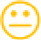	NR	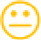							8/14(57%)
Zhang et al. (2020)	4	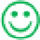	NR	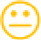	20–30 min	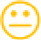									4	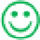	NR	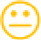	NR	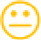	8/12(67%)
Zhang et al. (2015)	5	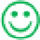	NR	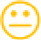	40 min	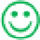	3	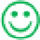	NR	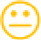	NR	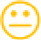	NR	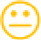							10/14(71%)
Li et al. (2022b)	5	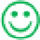	HRmax: 60%–75%	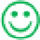	40–50 min	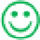															6/6(100%)
Yeh et al. (2021)	3	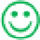	from the initial 40%–50% gradually increased to 60%–70% HRmax	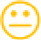	30 min	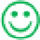	3	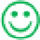	NR	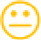	NR	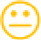	NR	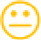							10/14(71%)
Zheng et al. (2020)	3	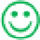	NR	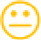	40 min	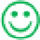															5/6(83%)

Note: ACSM, American College of Sports Medicine. Freq: frequency. VO_2_ R, oxygen uptake reserve. HRR, heart rate reserve. HRmax: maximal heart rate. RPE: ratings of perceived exertion. Ind. tail, individually tailored. NR, not reported. Happy/green face: meeting the criteria (2 points), neutral/yellow face: uncertain (1 point), unhappy/red face: not meeting the criteria (0 point).

### 3.4 Compliance with the ACSM recommendations

The intervention groups were categorized based on ACSM guidelines into two groups: those with high adherence and those with low or uncertain adherence. Among the ten studies, exercise interventions adhered to the ACSM recommendations with a compliance ratio of ≥75%. In contrast, in eight studies, exercise interventions exhibited a compliance ratio of <75% (refer to Table 4). The insufficient adherence (<75%) to the recommended prescription can be partly attributed to the failure of experimental designs to encompass all recommended parameters. Moreover, inadequate information on exercise prescribing hindered proper evaluation.

### 3.5 Meta-analyses

**FIGURE 4:**
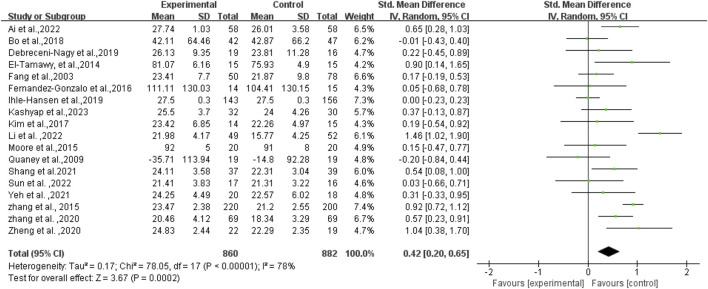
Forest plot of the effect of exercise on cognitive function in patients with PSCI.

Initially, an overall heterogeneity test was performed on the included literature, revealing high heterogeneity among multiple studies (*I* ^2^ = 78%, *p* < 0.1). Consequently, a random-effects model was employed for statistical analyses. Data analyses revealed that the experimental group exhibited higher cognitive function than the control group, with an overall SMD of 0.42 (95% CI: 0.20, 0.65). These results demonstrated a statistically significant difference (*p* < 0.01), as illustrated in Figure 4.

**FIGURE 5:**
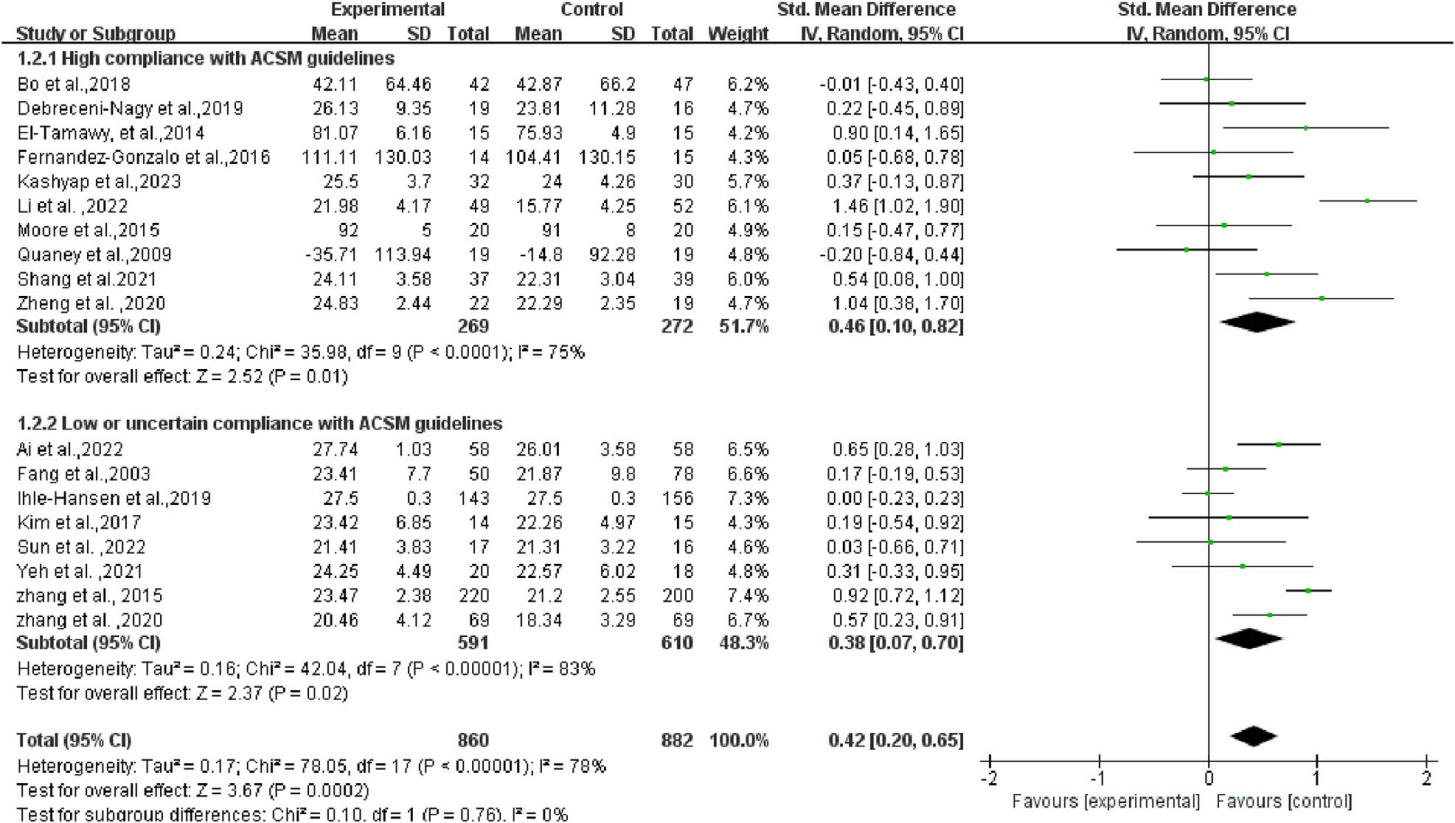
Subgroup analysis of the effect of exercise on cognitive function in patients with PSCI.

In a more detailed analysis, subgroups were examined based on high and low or uncertain adherence to ACSM recommendations. The research results are as follows: The SMD of the group with high compliance to ACSM guidelines was 0.46 (95% CI: 0.10, 0.82) (*p* = 0.01), with a heterogeneity of 75%, suggesting that engaging in exercise with a high adherence to ACSM recommendations potentially enhances cognitive function in individuals with PSCI. The SMD of the group with low or uncertain compliance to ACSM guidelines was 0.38 (95% CI: 0.07, 0.70) (*p* = 0.02), with a high heterogeneity of 83%. This finding reveals that exercise with low or uncertain compliance to ACSM guidelines also has a positive impact on cognition in individuals with PSCI. However, the high heterogeneity indicates potential regulatory variables among these studies. When comparing exercise with low/uncertain compliance to exercise with high compliance, the latter shows a stronger correlation with cognition in individuals with PSCI (SMD: high compliance 0.46 > low/uncertain compliance 0.38) (*p* < 0.01) (as illustrated in Figure 5).

**FIGURE 6:**
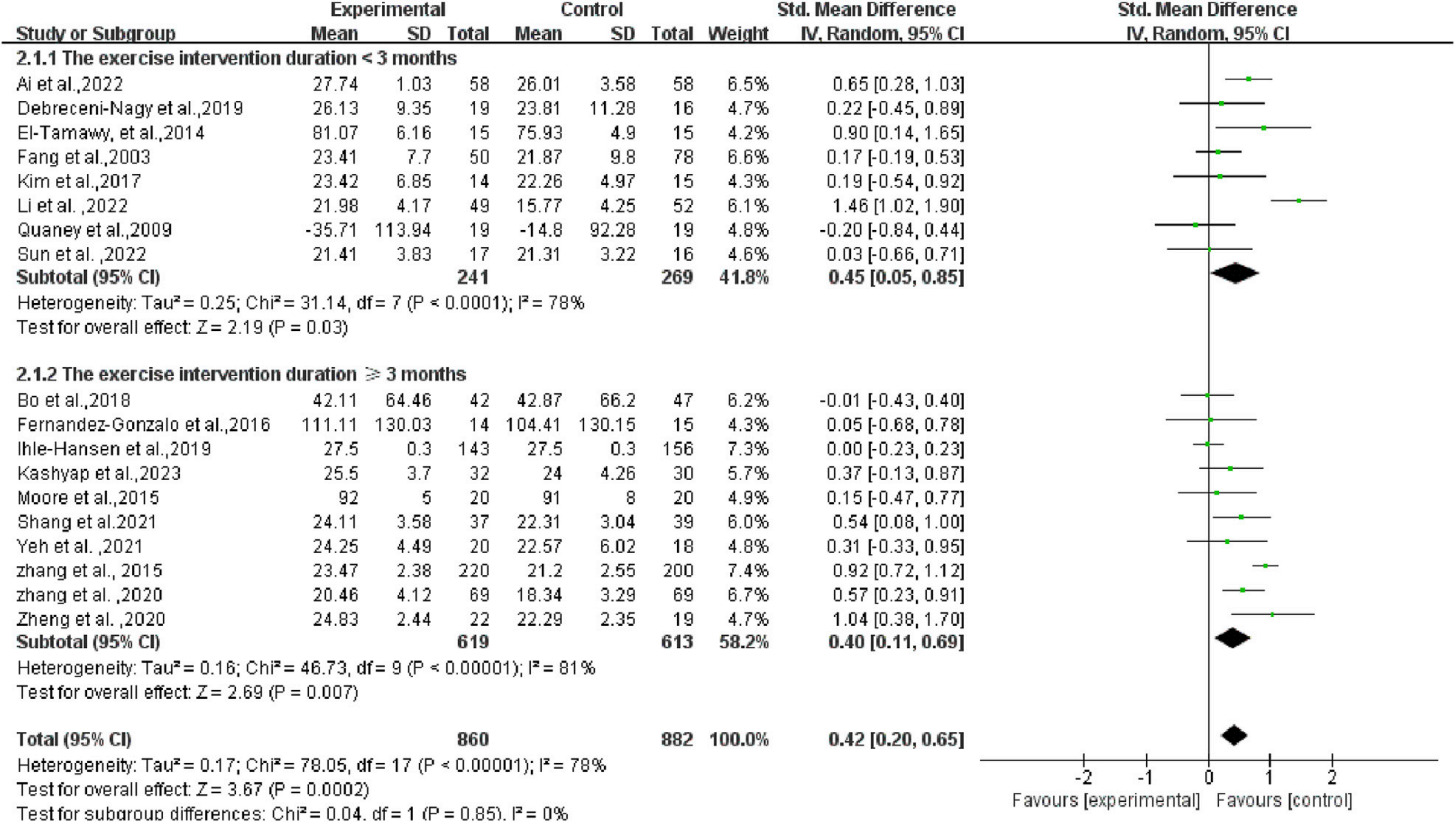
Subgroup analysis of the duration of the exercise intervention in patients with PSCI.

As the duration of the exercise intervention spanned a large period of time (from 4 to 18 months), subgroup analyses of the duration of the programs were also conducted. The research results are as follows: The SMD of the group with the exercise intervention duration <3 months was 0.45 (95% CI: 0.05, 0.85) (*p* = 0.03), and the SMD of the group with the exercise intervention duration ≥3 months was 0.40 (95% CI: 0.11, 0.69) (*p* = 0.007). There was no statistical difference between the two groups (*p* = 0.85 > 0.05) (as illustrated in Figure 6). This result indicates that the duration of the programs did not significantly influence our overall survey findings.

**FIGURE 7:**
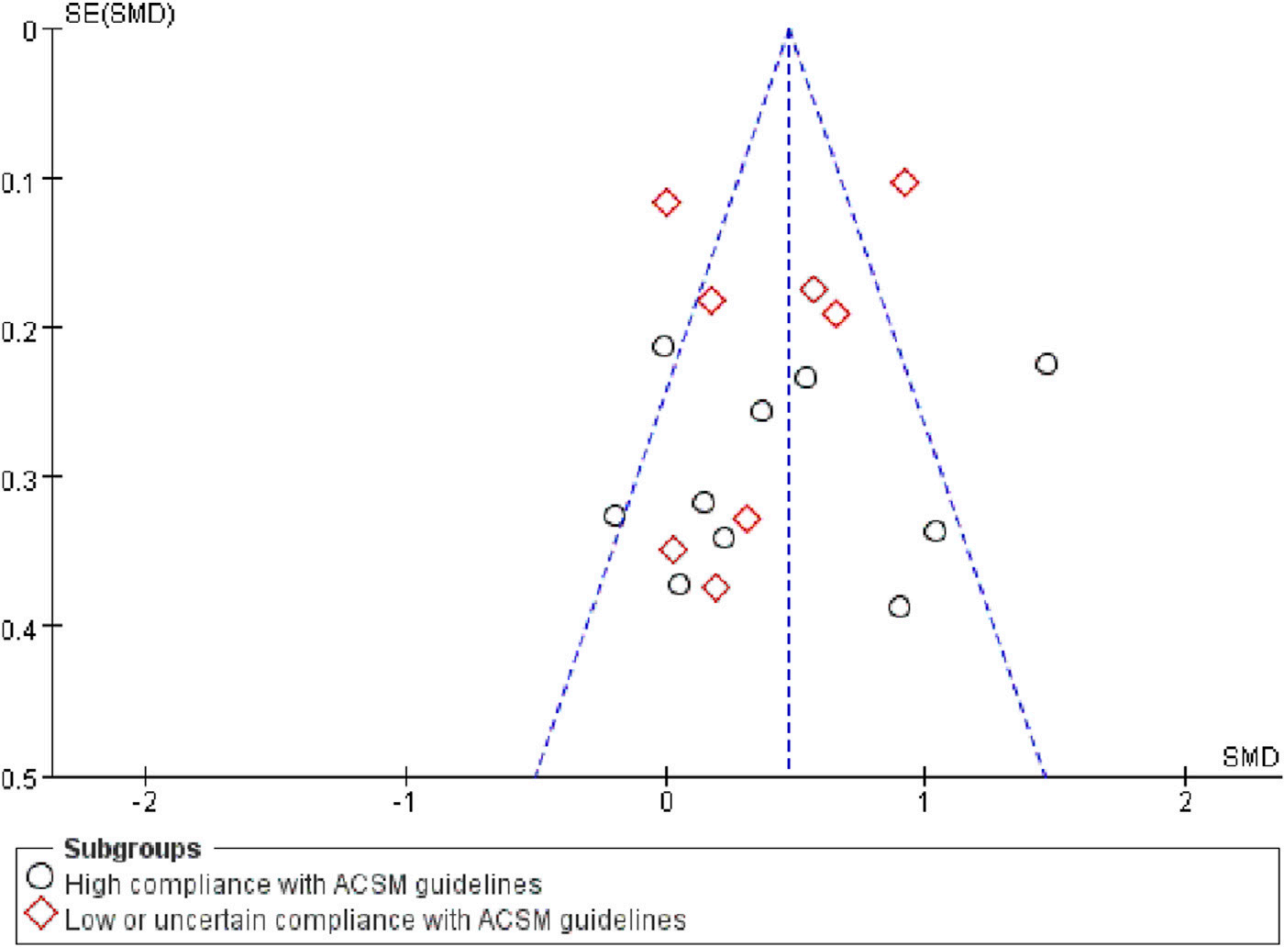
Funnel plot of the included study.

Finally, tests were conducted to detect publication bias. Upon examining the funnel plot (Figure 7), we observed a close balance in the distribution of studies on both sides, suggesting no significant publication bias.

## 4 Discussion

In this study, we investigated the impact of different exercise doses on cognitive function in patients with PSCI. Our analysis was based on 18 studies which included a total of 1,742 participants. The results revealed that exercise intervention can enhance cognitive function in individuals with PSCI, aligning with previous research conclusions (Li et al., 2023; Zhang et al., 2023).

Previous studies have established that exercise, as a non-drug therapy, can be effective in improving cognitive function in patients with PSCI (Lanctôt et al., 2020). However, the effectiveness varies among different exercise interventions. Meta-analyses by Zhang et al. and Li et al. demonstrated that aerobic exercise, resistance exercise, or a combination of both had positive effects on cognitive function of patients with PSCI. Aerobic exercise particularly showed a significant clinical impact on enhancing cognitive function, while the other two exercise types exhibited moderate effects (Zhang et al., 2023). Lightweight and operable aerobic exercises were emphasized as having significant positive impacts on alleviating cognitive impairments after stroke (Li et al., 2023).

Exercise contributes to cognitive improvement through various mechanisms. First, it has the potential to enhance cardiorespiratory functionality, reduce brain atrophy, increase cerebral blood flow, facilitate neural connections, boost brain tissue metabolism, and stimulate excitation in the central nervous system (Szulc-Lerch et al., 2018). Second, by actively modifying vascular risk factors, exercise plays a pivotal role in preventing stroke, a key aspect of managing PSCI (Al Jerdi et al., 2020). Evidence suggests that exercise is effective in reducing risk factors for stroke, such as diabetes mellitus, coronary heart disease, high cholesterol, and hypertension, all of which contribute to vascular cognitive deterioration (Sahathevan et al., 2012). Additionally, exercise may enhance cognitive function by increasing the expression of growth factors like brain-derived neurotrophic factor, insulin-like growth factor-1, and vascular endothelial growth factor, thereby facilitating neurogenesis and angiogenesis (Cotman et al., 2007; Winter et al., 2007).

Beyond considering the type of exercise, it is crucial to examine the impact of varying exercise dosages on cognitive function in patients with PSCI. Existing evidence suggests that moderate-intensity exercise has the best effect on global cognition in patients with cognitive dysfunction after stroke, followed by low-intensity exercise (Li X. et al., 2022a). Initiating light-to-moderate intensity exercise early after a stroke may particularly improve memory, learning, and attention (Hasan et al., 2016). Recommendations from the American Academy of Neurology propose that engaging in exercise twice per week could potentially benefit cognitive function in individuals with cognitive dysfunction (Petersen et al., 2018). However, recent studies have shown mixed results, with some indicating no significant improvements in cognitive function with certain exercise intensities (Lapointe et al., 2022). Consequently, there remains a gap in research on exercise dosage for patients with PSCI, highlighting the need for further exploration of exercise intensity, frequency, total duration, and session duration.

In this comprehensive review, we synthesized information from various studies, incorporating different types of exercises, intensity levels, frequencies, durations, and other relevant indicators. Our analysis involved calculating exercise adherence scores based on ACSM recommendations, dividing the participants into a high compliance group and a low or uncertain compliance group based on scoring ratios. Subgroup analyses were then conducted on both groups to explore the impact of exercise dose on enhancing cognitive function in patients with PSCI.

The results from the subgroup analysis revealed a significant positive effect of exercise on cognitive function in both the high adherence and low/uncertain adherence groups [high SMD = 0.46, 95% CI (0.10, 0.82), low or uncertain SMD = 0.38, 95% CI (0.07, 0.70)], with statistical significance (*p* < 0.05). This suggests that exercise is beneficial for patients with PSCI over no exercise, irrespective of whether the exercise dose is highly compliant with ACSM recommendations. This finding aligns with the notion that exercise itself holds a high potential for improving cognitive function after a stroke (Li et al., 2023). However, the key difference in our study is the observation that high compliance with the exercise dose is more advantageous for enhancing cognitive function than low or uncertain compliance (high SMD = 0.46 > low or uncertain SMD = 0.38).

Examining the exercise types within the two groups, the ACSM high-compliance group involved various modalities such as aerobic training, physical exercise, resistance training, yoga, strength training, and eight-segment exercise training. Similarly, the ACSM low or uncertain adherence group included activities like walking, aerobic exercise, physical activity, passive movement training, resistance training, and strength training. Notably, the study aimed to mitigate the influence of major exercise types on differences in ACSM compliance. The primary recommended exercises in the ACSM guidelines encompass resistance exercise, aerobic exercise, and flexibility exercise, accompanied by detailed dosage recommendations for each modality. However, the majority of the literature included in the analysis focused on either a singular type of exercise or a combination of two types of training. Moreover, the descriptions of exercise dosage in the literature lack comprehensiveness. While some studies mentioned the mode and total duration of the exercise intervention, there was a notable absence or insufficient reporting on the intensity and frequency of the intervention. The term “individualized or individually tailored” was predominantly used to describe the exercise dose. Consequently, studies that should have been categorized as high adherence were mistakenly labeled as low or uncertain compliance. This misclassification may have influenced the overall findings and interpretations.

In clinical practice, it is essential to customize the exercise intervention dosage based on ACSM guidelines to provide personalized exercise regimens for patients with PSCI. The findings of this study emphasize the significant advantages linked to high adherence to the exercise dose recommended by ACSM guidelines compared to low or uncertain adherence. These results have promising clinical implications and serve as a benchmark for creating standardized and systematic exercise intervention programs. Therefore, we advocate for the early initiation of an appropriate exercise program for patients with PSCI under professional guidance. During exercise treatment, adjustments to the exercise dose should be made based on individual fitness, with a gradual strengthening of the dose while ensuring patient safety until it aligns with high compliance with ACSM recommendations. Future research should include additional RCTs with larger samples, multiple centers, and more rigorous research designs following the ACSM guidelines. This will help confirm our findings and establish a more scientific and reliable foundation for developing systematic, standardized, and repeatable exercise intervention prescriptions.

## 5 Strengths and limitations

This article marks attempt to assess the impact of various exercise doses on cognitive function in individuals with PSCI based on the ACSM recommendations. As such, it introduces a topic with significant clinical implications. The research methodology employed is robust, featuring a comprehensive and rigorous search strategy. Additionally, a dual review process was implemented to uphold the accuracy and reliability of the research. The inclusion of only RCTs further enhances the quality of the evidence synthesized.

However, the study is not without limitations. Firstly, the pool of eligible RCTs addressing the influence of exercise on patients with PSCI remains limited. This scarcity underscores the need for more relevant studies in the future to expand the knowledge base in this domain. Secondly, the study does not consider several factors, including stroke timing, severity, subtype, infarction site, and individual characteristics such as age, sex, or other baseline factors. The omission of these variables may contribute to inevitable heterogeneity among the included studies. Additionally, while we mentioned the significance of motor-cognitive dual-task training in patient rehabilitation in the introduction, this aspect is not explored in detail within the main scope of our study, which focuses on determining the optimal exercise dosage for patients. Future research should consider conducting comprehensive studies on dual-task training to evaluate whether a combination of exercise and cognitive training offers greater benefits for PSCI patients and to investigate the underlying mechanisms. Lastly, despite promising findings, the results should be interpreted cautiously due to the observed high heterogeneity among the studies.

## 6 Conclusion

In our study, we observed a significant positive impact of exercise on cognitive function in patients with PSCI. Importantly, our findings indicate that high adherence to the exercise dose recommended by the ACSM guidelines had a more substantial effect on cognitive function in patients with PSCI than low or uncertain compliance to ACSM recommendations. This underscores the potential benefits of closely following the prescribed exercise guidelines for individuals dealing with PSCI.

## Data Availability

The original contributions presented in the study are included in the article/Supplementary Material, further inquiries can be directed to the corresponding author.

## References

[B1] Ai W. P. Guo Z. F. Zhang X. J. Zhang J. (2022). Efficacy of anti-resistance training combined with computerized cognitive training for mild cognitive impairment after stroke. Chin. J. Rehabil. 37, 456–459. 10.3870/zgkf.2022.08.002

[B2] Al Jerdi S. Aleyadeh R. Imam Y. (2020). Management of cognitive impairment after stroke. Curr. Treat. Options Neurology 22, 20. 10.1007/s11940-020-00627-3

[B3] Atteih S. Mellon L. Hall P. Brewer L. Horgan F. Williams D. (2015). Implications of stroke for caregiver outcomes: findings from the ASPIRE-S study. Int. J. Stroke 10, 918–923. 10.1111/ijs.12535 26061711

[B4] Battle C. E. Abdul-Rahim A. H. Shenkin S. D. Hewitt J. Quinn T. J. (2021). Cholinesterase inhibitors for vascular dementia and other vascular cognitive impairments: a network meta-analysis. Cochrane Database Syst. Rev. 2, Cd013306. 10.1002/14651858.CD013306.pub2 33704781 PMC8407366

[B5] Bo W. Lei M. Tao S. Jie L. T. Qian L. Lin F. Q. (2019). Effects of combined intervention of physical exercise and cognitive training on cognitive function in stroke survivors with vascular cognitive impairment: a randomized controlled trial. Clin. Rehabil. 33, 54–63. 10.1177/0269215518791007 30064268

[B6] Chang Y. K. Chu C. H. Wang C. C. Wang Y. C. Song T. F. Tsai C. L. (2015). Dose-response relation between exercise duration and cognition. Med. Sci. Sports Exerc 47, 159–165. 10.1249/mss.0000000000000383 24870572

[B7] Cotman C. W. Berchtold N. C. Christie L. A. (2007). Exercise builds brain health: key roles of growth factor cascades and inflammation. Trends Neurosci. 30, 464–472. 10.1016/j.tins.2007.06.011 17765329

[B8] Debreceni-Nagy A. Horváth J. Bajuszné Kovács N. Fülöp P. Jenei Z. (2019). The effect of low-intensity aerobic training on cognitive functions of severely deconditioned subacute and chronic stroke patients: a randomized, controlled pilot study. Int. J. Rehabil. Res. 42, 275–279. 10.1097/mrr.0000000000000346 30882527

[B9] Ding M. Y. Xu Y. Wang Y. Z. Li P. X. Mao Y. T. Yu J. T. (2019). Predictors of cognitive impairment after stroke: a prospective stroke cohort study. J. Alzheimers Dis. 71, 1139–1151. 10.3233/jad-190382 31524163

[B10] El-Tamawy M. S. Abd-Allah F. Ahmed S. M. Darwish M. H. Khalifa H. A. (2014). Aerobic exercises enhance cognitive functions and brain derived neurotrophic factor in ischemic stroke patients. NeuroRehabilitation 34, 209–213. 10.3233/nre-131020 24284463

[B11] Fang Y. Chen X. Li H. Lin J. Huang R. Zeng J. (2003). A study on additional early physiotherapy after stroke and factors affecting functional recovery. Clin. Rehabil. 17, 608–617. 10.1191/0269215503cr655oa 12971705

[B12] Fernandez-Gonzalo R. Fernandez-Gonzalo S. Turon M. Prieto C. Tesch P. A. García-Carreira Mdel C. (2016). Muscle, functional and cognitive adaptations after flywheel resistance training in stroke patients: a pilot randomized controlled trial. J. Neuroeng Rehabil. 13, 37. 10.1186/s12984-016-0144-7 27052303 PMC4823904

[B13] Garber C. E. Blissmer B. Deschenes M. R. Franklin B. A. Lamonte M. J. Lee I. M. (2011). American College of Sports Medicine position stand. Quantity and quality of exercise for developing and maintaining cardiorespiratory, musculoskeletal, and neuromotor fitness in apparently healthy adults: guidance for prescribing exercise. Med. Sci. Sports Exerc 43, 1334–1359. 10.1249/MSS.0b013e318213fefb 21694556

[B14] Hasan S. M. Rancourt S. N. Austin M. W. Ploughman M. (2016). Defining optimal aerobic exercise parameters to affect complex motor and cognitive outcomes after stroke: a systematic review and synthesis. Neural Plast. 2016, 2961573. 10.1155/2016/2961573 26881101 PMC4736968

[B15] He A. Wang Z. Wu X. Sun W. Yang K. Feng W. (2023). Incidence of post-stroke cognitive impairment in patients with first-ever ischemic stroke: a multicenter cross-sectional study in China. Lancet Reg. Health West Pac 33, 100687. 10.1016/j.lanwpc.2023.100687 37181529 PMC10166998

[B16] Hernandez A. G. Gonzalez-Galvez N. (2021). Ejercicio físico y función cognitiva en pacientes postictus: una revisión sistemática con metaanálisis. APUNTS Educ. Fis. Y Deport., 1–10. 10.5672/apunts.2014-0983.es.(2021/4).146.01

[B17] Higgins J. P. Thompson S. G. Deeks J. J. Altman D. G. (2003). Measuring inconsistency in meta-analyses. Bmj 327, 557–560. 10.1136/bmj.327.7414.557 12958120 PMC192859

[B18] Huang X. Zhao X. Li B. Cai Y. Zhang S. Wan Q. (2022). Comparative efficacy of various exercise interventions on cognitive function in patients with mild cognitive impairment or dementia: a systematic review and network meta-analysis. J. Sport Health Sci. 11, 212–223. 10.1016/j.jshs.2021.05.003 34004389 PMC9068743

[B19] Ihle-Hansen H. Langhammer B. Lydersen S. Gunnes M. Indredavik B. Askim T. (2019). A physical activity intervention to prevent cognitive decline after stroke: secondary results from the Life after STroke study, an 18-month randomized controlled trial. J. Rehabil. Med. 51, 646–651. 10.2340/16501977-2588 31440765

[B20] Kashyap M. Rai N. K. Singh R. Joshi A. Rozatkar A. R. Kashyap P. V. (2023). Effect of early yoga practice on post stroke cognitive impairment. Ann. Indian Acad. Neurol. 26, 59–66. 10.4103/aian.aian_808_22 37034037 PMC10081555

[B21] Kernan W. N. Viera A. J. Billinger S. A. Bravata D. M. Stark S. L. Kasner S. E. (2021). Primary care of adult patients after stroke: a scientific statement from the American heart association/American stroke association. Stroke 52, e558–e571. 10.1161/str.0000000000000382 34261351

[B22] Kim G. Y. Han M. R. Lee H. G. (2014). Effect of dual-task rehabilitative training on cognitive and motor function of stroke patients. J. Phys. Ther. Sci. 26, 1–6. 10.1589/jpts.26.1 24567664 PMC3927016

[B23] Kim J. Yim J. (2017). Effects of an exercise protocol for improving handgrip strength and walking speed on cognitive function in patients with chronic stroke. Med. Sci. Monit. 23, 5402–5409. 10.12659/msm.904723 29131814 PMC5699168

[B24] Koton S. Pike J. R. Johansen M. Knopman D. S. Lakshminarayan K. Mosley T. (2022). Association of ischemic stroke incidence, severity, and recurrence with dementia in the atherosclerosis risk in communities cohort study. JAMA Neurol. 79, 271–280. 10.1001/jamaneurol.2021.5080 35072712 PMC8787684

[B25] Lanctôt K. L. Lindsay M. P. Smith E. E. Sahlas D. J. Foley N. Gubitz G. (2020). Canadian stroke best practice recommendations: mood, cognition and fatigue following stroke, 6th edition update 2019. Int. J. Stroke 15, 668–688. 10.1177/1747493019847334 31221036

[B26] Lapointe T. Houle J. Sia Y. T. Payette M. Trudeau F. (2022). Addition of high-intensity interval training to a moderate intensity continuous training cardiovascular rehabilitation program after ischemic cerebrovascular disease: a randomized controlled trial. Front. Neurol. 13, 963950. 10.3389/fneur.2022.963950 36686521 PMC9846748

[B27] Li W. Luo Z. Jiang J. Li K. Wu C. (2023). The effects of exercise intervention on cognition and motor function in stroke survivors: a systematic review and meta-analysis. Neurol. Sci. 44, 1891–1903. 10.1007/s10072-023-06636-9 36781567

[B28] Li X. Geng D. Wang S. Sun G. (2022a). Aerobic exercises and cognitive function in post-stroke patients: a systematic review with meta-analysis. Med. Baltim. 101, e31121. 10.1097/md.0000000000031121 PMC9575743 36253969

[B29] Li Y. Yang L. Zhu S. M. (2022b). Effect of aerobic exercise combined with hyperbaric oxygen on cognitive dysfunction after stroke and its effect on oxidative stress. J. Med. Inf. 35, 114–116. 10.3969/j.issn.1006-1959.2022.17.030

[B30] Liu Y. Chen F. Qin P. Zhao L. Li X. Han J. (2023). Acupuncture treatment vs. cognitive rehabilitation for post-stroke cognitive impairment: a systematic review and meta-analysis of randomized controlled trials. Front. Neurol. 14, 1035125. 10.3389/fneur.2023.1035125 36846126 PMC9946978

[B31] Mijajlović M. D. Pavlović A. Brainin M. Heiss W. D. Quinn T. J. Ihle-Hansen H. B. (2017). Post-stroke dementia - a comprehensive review. BMC Med. 15, 11. 10.1186/s12916-017-0779-7 28095900 PMC5241961

[B32] Moore S. A. Hallsworth K. Jakovljevic D. G. Blamire A. M. He J. Ford G. A. (2015). Effects of community exercise therapy on metabolic, brain, physical, and cognitive function following stroke: a randomized controlled pilot trial. Neurorehabil Neural Repair 29, 623–635. 10.1177/1545968314562116 25538152

[B33] Narasimhalu K. Effendy S. Sim C. H. Lee J. M. Chen I. Hia S. B. (2010). A randomized controlled trial of rivastigmine in patients with cognitive impairment no dementia because of cerebrovascular disease. Acta Neurol. Scand. 121, 217–224. 10.1111/j.1600-0404.2009.01263.x 19951274

[B34] Obaid M. Douiri A. Flach C. Prasad V. Marshall I. (2020). Can we prevent poststroke cognitive impairment? An umbrella review of risk factors and treatments. BMJ Open 10, e037982. 10.1136/bmjopen-2020-037982 PMC7482478 32912953

[B35] Page M. J. Mckenzie J. E. Bossuyt P. M. Boutron I. Hoffmann T. C. Mulrow C. D. (2021). The PRISMA 2020 statement: an updated guideline for reporting systematic reviews. Bmj 372, n71. 10.1136/bmj.n71 33782057 PMC8005924

[B36] Petersen R. C. Lopez O. Armstrong M. J. Getchius T. S. D. Ganguli M. Gloss D. (2018). Practice guideline update summary: mild cognitive impairment: report of the guideline development, dissemination, and implementation subcommittee of the American Academy of Neurology. Neurology 90, 126–135. 10.1212/wnl.0000000000004826 29282327 PMC5772157

[B37] Quaney B. M. Boyd L. A. Mcdowd J. M. Zahner L. H. He J. Mayo M. S. (2009). Aerobic exercise improves cognition and motor function poststroke. Neurorehabil Neural Repair 23, 879–885. 10.1177/1545968309338193 19541916 PMC3024242

[B38] Quinn T. J. Richard E. Teuschl Y. Gattringer T. Hafdi M. O'brien J. T. (2021). European Stroke Organisation and European Academy of Neurology joint guidelines on post-stroke cognitive impairment. Eur. J. Neurol. 28, 3883–3920. 10.1111/ene.15068 34476868

[B39] Rost N. S. Brodtmann A. Pase M. P. Van Veluw S. J. Biffi A. Duering M. (2022). Post-stroke cognitive impairment and dementia. Circ. Res. 130, 1252–1271. 10.1161/circresaha.122.319951 35420911

[B40] Sahathevan R. Brodtmann A. Donnan G. A. (2012). Dementia, stroke, and vascular risk factors; a review. Int. J. Stroke 7, 61–73. 10.1111/j.1747-4949.2011.00731.x 22188853

[B41] Shang X. Meng X. Xiao X. Xie Z. Yuan X. (2021). Grip training improves handgrip strength, cognition, and brain white matter in minor acute ischemic stroke patients. Clin. Neurol. Neurosurg. 209, 106886. 10.1016/j.clineuro.2021.106886 34455171

[B42] Sun J. H. Tan L. Yu J. T. (2014). Post-stroke cognitive impairment: epidemiology, mechanisms and management. Ann. Transl. Med. 2, 80. 10.3978/j.issn.2305-5839.2014.08.05 25333055 PMC4200648

[B43] Sun R. Li X. Zhu Z. Li T. Zhao M. Mo L. (2022). Effects of dual-task training in patients with post-stroke cognitive impairment: a randomized controlled trial. Front. Neurol. 13, 1027104. 10.3389/fneur.2022.1027104 36353135 PMC9639668

[B44] Szulc-Lerch K. U. Timmons B. W. Bouffet E. Laughlin S. De Medeiros C. B. Skocic J. (2018). Repairing the brain with physical exercise: cortical thickness and brain volume increases in long-term pediatric brain tumor survivors in response to a structured exercise intervention. Neuroimage Clin. 18, 972–985. 10.1016/j.nicl.2018.02.021 29876282 PMC5987848

[B45] Winter B. Breitenstein C. Mooren F. C. Voelker K. Fobker M. Lechtermann A. (2007). High impact running improves learning. Neurobiol. Learn Mem. 87, 597–609. 10.1016/j.nlm.2006.11.003 17185007

[B46] Yeh T. T. Chang K. C. Wu C. Y. Chen C. J. Chuang I. C. (2022). Clinical efficacy of aerobic exercise combined with computer-based cognitive training in stroke: a multicenter randomized controlled trial. Top. Stroke Rehabil. 29, 255–264. 10.1080/10749357.2021.1922045 34340637

[B47] Zhang J. Lyu D. Li J. (2015). The impact of aerobic combined strength and balance exercise on cognitive function in patients with cognitive impairment no dementia. Chin. J. Prac. Nurs. 31, 2435–2438. 10.3760/cma.j.issn.1672-7088.2015.32.005

[B48] Zhang X. Bi X. (2020). Post-stroke cognitive impairment: a review focusing on molecular biomarkers. J. Mol. Neurosci. 70, 1244–1254. 10.1007/s12031-020-01533-8 32219663

[B49] Zhang X. X. Zheng Y. Ma L. Xu Y. Q. Zhang H. M. Li H. Y. (2020). Effects of aerobic exercise training on insulin resistance and cognitive level in stroke patients. Chin. J. Med. 55, 434–437. 10.3969/j.issn.1008-1070.2020.04.026

[B50] Zhang Y. Qiu X. Chen J. Ji C. Wang F. Song D. (2023). Effects of exercise therapy on patients with poststroke cognitive impairment: a systematic review and meta-analysis. Front. Neurosci. 17, 1164192. 10.3389/fnins.2023.1164192 37090811 PMC10117650

[B51] Zheng G. Zheng Y. Xiong Z. Ye B. (2020). Effect of Baduanjin exercise on cognitive function in patients with post-stroke cognitive impairment: a randomized controlled trial. Clin. Rehabil. 34, 1028–1039. 10.1177/0269215520930256 32517490

